# Engineering intracellular malonyl-CoA availability in microbial hosts and its impact on polyketide and fatty acid synthesis

**DOI:** 10.1007/s00253-020-10643-7

**Published:** 2020-05-08

**Authors:** Lars Milke, Jan Marienhagen

**Affiliations:** 1 Institute of Bio- and Geosciences, IBG-1: Biotechnology, Forschungszentrum Jülich GmbH, 52425 Jülich, Germany; 2 Institute of Biotechnology, RWTH Aachen University, Worringer Weg 3, 52074 Aachen, Germany; 3 Bioeconomy Science Center (BioSC), Forschungszentrum Jülich GmbH, 52425 Jülich, Germany

**Keywords:** Malonyl-CoA, Metabolic engineering, Polyketide, Fatty acid, Biofuel

## Abstract

Malonyl-CoA is an important central metabolite serving as the basic building block for the microbial synthesis of many pharmaceutically interesting polyketides, but also fatty acid–derived compounds including biofuels. Especially *Saccharomyces cerevisiae*, *Escherichia coli*, and *Corynebacterium glutamicum* have been engineered towards microbial synthesis of such compounds in recent years. However, developed strains and processes often suffer from insufficient productivity. Usually, tightly regulated intracellular malonyl-CoA availability is regarded as the decisive bottleneck limiting overall product formation. Therefore, metabolic engineering towards improved malonyl-CoA availability is essential to design efficient microbial cell factories for the production of polyketides and fatty acid derivatives. This review article summarizes metabolic engineering strategies to improve intracellular malonyl-CoA formation in industrially relevant microorganisms and its impact on productivity and product range, with a focus on polyketides and other malonyl-CoA-dependent products.

Key Points

• *Malonyl-CoA is the central building block of polyketide synthesis.*

• *Increasing acetyl-CoA supply is pivotal to improve malonyl-CoA availability.*

• *Improved acetyl-CoA carboxylase activity increases availability of malonyl-CoA.*

• *Fatty acid synthesis as an ambivalent target to improve malonyl-CoA supply.*

## Introduction

**Fig. 1:**
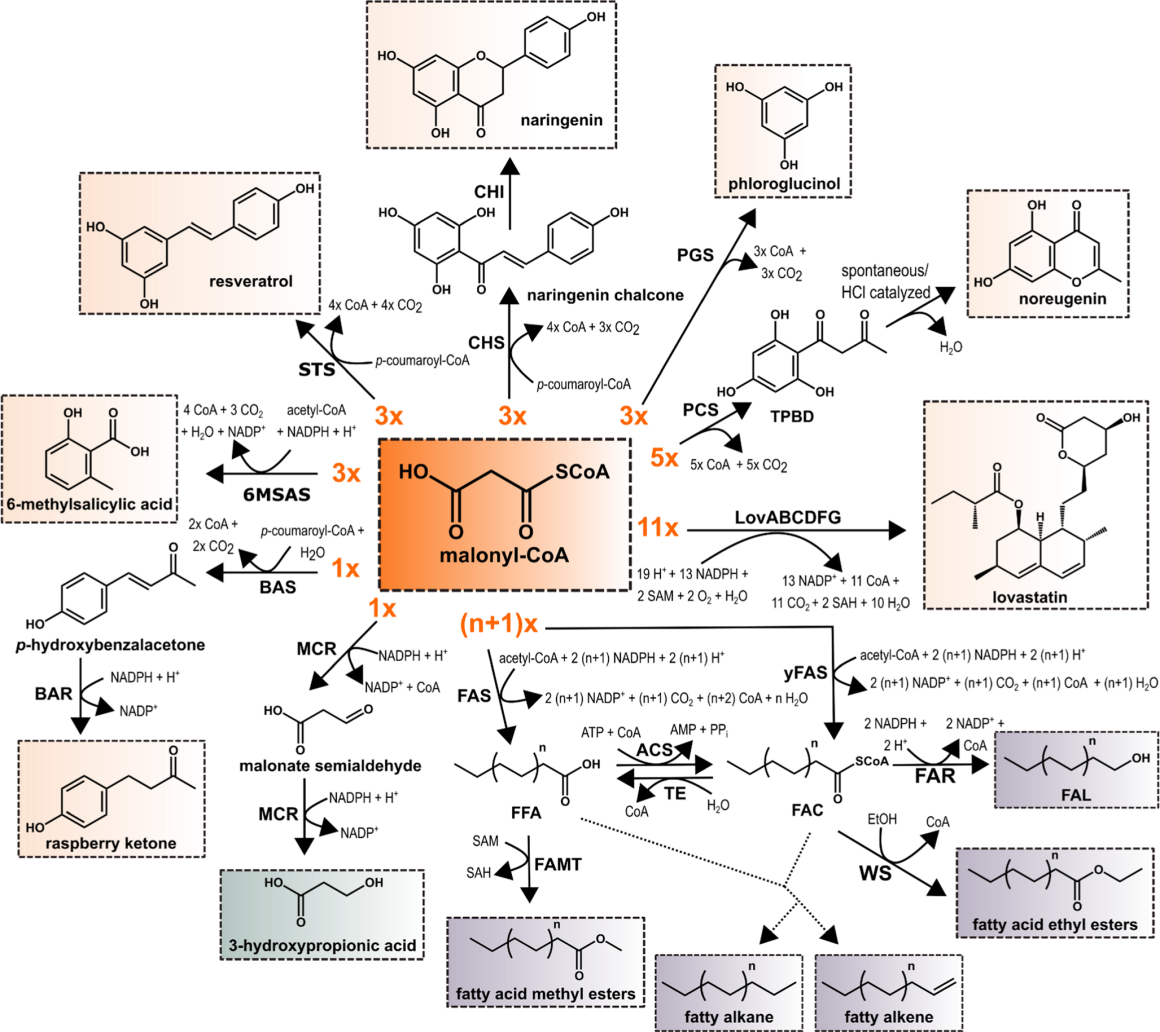
Overview of different microbially synthesized malonyl-CoA-dependent molecules. 6MSAS 6-methylsalicylic acid synthase, ACS acyl-CoA synthetase, BAR benzalacetone reductase, BAS benzalacetone synthase, CHI chalcone isomerase, CHS chalcone synthase, FAC fatty-acyl-CoA, FAL fatty acid alcohol, FAMT fatty acid *O*-methyltransferase, FAR fatty-acyl-CoA reductase, FAS fatty acid synthase, FFA free fatty acid, LovABCDFG lovastatin megasynthase complex from *Aspergillus terreus*, MCR malonyl-CoA reductase, PCS pentaketide chromone synthase, PGS phloroglucinol synthase, SAH *S*-adenosyl-homocysteine, SAM *S*-adenosylmethionine, STS stilbene synthase, TE fatty-acyl-CoA thioesterase, TPBD 1-(2,4,6-tri-hydroxyphenyl)butane-1,3-dione, WS wax ester synthase, yFAS yeast fatty acid synthase/fatty-acyl-CoA synthase. Light orange boxes indicate polyketide products, light green boxes carboxylic acids, and light purple boxes fatty acid–derived biofuels. For fatty acid synthesis, *n* represents the number of malonyl-CoA-derived C2 units incorporated into the growing acyl-chain between the acetyl-CoA-derived starter unit (terminal methyl group) and the final malonyl-CoA-derived extender unit (terminal carboxyl group), which accounts for *+* 1

Polyketides are an outstanding group of secondary metabolites with regard to their structural diversity and the number of their clinical applications (Hopwood 2009; Osbourn and Lanzotti 2009; Wink 2010; Robertsen and Musiol-Kroll 2019). Not only does this group comprise antibiotics (e.g., erythromycin A, azithromycin), anticancer drugs (e.g., enediynes), and drugs for the treatment of cardiovascular diseases (e.g., lovastatin) but also important immunosuppressants such as rapamycin. Of the 7,000 polyketides known, more than 20 have been commercialized, which resembles a “success rate” of 0.3% (Weissman and Leadlay 2005). In total, polyketide-derived pharmaceuticals make up 20% of the top-selling drugs, generating a worldwide revenue of over €14 billion annually (Weissman and Leadlay 2005). Additionally, polyketides include polyphenols comprising flavonoids and stilbenes such as naringenin and resveratrol (Fig. 1). These molecules provide a plethora of beneficial effects on human health including antioxidant, anti-inflammatory, or anti-cancerous characteristics (Pandey and Rizvi 2009). Furthermore, a positive effect in prevention or treatment of cardiovascular and neurodegenerative diseases, but also obesity and diabetes, was described for selected compounds (Khurana et al. 2013). In this context, resveratrol is probably the most prominent example, which is successfully marketed as dietary supplement (Catalgol et al. 2012). In total, the global nutraceutical market was accounted for $379 billion in 2017 (Stratistics Market Research Consulting Pvt Ltd 2018).

Polyketides are ubiquitous metabolites found in bacteria, fungi, and plants. Despite their structural diversity, all polyketides are synthesized by iterative, decarboxylative Claisen condensation of acyl-CoA units catalyzed by polyketide synthases (PKSs). Based on their overall architectural structure, which defines starter unit selection, chain elongation, degree of reduction, and cyclization patterns, PKS are classified into three types (types I–III) (Austin and Noel 2003). Whereas the type I family comprises large, multifunctional polypeptides with multiple, catalytically active domains, type II family PKSs typically are aggregates of dissociable, monofunctional enzymes (Hertweck 2009). Depending on the PKS type, the individual domains or the monofunctional enzymes provide acyltransferase, acyl carrier protein, β-ketosynthase (KS), and thioesterase functions. β-keto processing capabilities including dehydratase, enoylreductase, and ketoreductase activities are optional. In accordance with the fatty acid synthase (FAS) classification, type I PKSs predominantly occur in fungi and animals, whereas type II PKSs are commonly found in prokaryotes, in particular actinomycetes. In contrast, type III PKSs—also referred to as chalcone/stilbene synthases (CHS/STS)—are KS-like homodimers usually to be found in plants, but also bacteria and some fungi. Despite their structural simplicity, type III PKSs can perform starter unit selection, catalyze (iterative) chain elongation, and control cyclization patterns. However, independent from this classification, most PKSs consume malonyl-CoA molecules as extender units, which are successively added to growing β-ketoacyl chains (Chan et al. 2009). Departing from this, the type I PKS from *Saccharopolyspora erythraea* synthesizing the 6-deoxyerythronolide B scaffold of erythromycin A condenses one propionyl-CoA starter unit with six methylmalonyl-CoA extender units (Rawlings 2001).

Depending on the respective polyketide product, very different malonyl-CoA quantities are required. Whereas the synthesis of the flavoring phenylbutanoid raspberry ketone requires only one malonyl-CoA molecule, three malonyl-CoA units are typically needed for the synthesis of plant polyphenols such as resveratrol and naringenin, and during lovastatin biosynthesis eleven malonyl-CoA molecules are consumed (Fig. 1) (Campbell and Vederas 2010; Shimokawa et al. 2012; Milke et al. 2018). In most organisms, malonyl-CoA is exclusively synthesized by acetyl-CoA carboxylation catalyzed by acetyl-CoA carboxylases (ACC). This enzyme complex comprises three different domains catalyzing two distinct reaction steps (Cronan and Waldrop 2002). Initially, an ATP-dependent biotin carboxylase (BC) domain catalyzes the carboxylation of biotin with bicarbonate, which is covalently attached to a biotin carboxyl carrier protein (BCCP) domain over a lysine residue, forming carboxybiotin. A flexible biotin arm shuttles the carboxybiotin from the BC domain to the carboxyltransferase (CT) domain required for the second half reaction in which the carboxygroup is transferred to acetyl-CoA forming malonyl-CoA. However, the role of malonyl-CoA as building block for secondary metabolite synthesis plays only a minor role in the cellular metabolism. Primarily, malonyl-CoA serves as extender unit for the synthesis of fatty acids, constituting the hydrophobic domain of membrane lipids (Cronan and Thomas 2009). Noteworthy, fatty acid–derived alcohols (FAL), alkyl esters (FAME, FAEE), and alkanes/alkenes are considered to be promising second-generation biofuels as they provide similar chemical properties as petroleum-based fuels, allowing to readily replace them (Fig. 1) (Sheng and Feng 2015; Hu et al. 2019). In total, the global market for natural fatty acids had an value of nearly $13.5 billion in 2018 (BCC Research LLC 2019).

Current efforts towards the transformation of a fossil-based economy to a more sustainable bio-based economy have drawn attention to the microbial synthesis of both polyketides and fatty acids (Takeno et al. 2013; Yang et al. 2018; Hu et al. 2019). Unfortunately, the strictly regulated intracellular malonyl-CoA availability in well-established microbial platform organisms was identified as decisive bottleneck limiting overall product formation (Marienhagen and Bott 2013; Janßen and Steinbüchel 2014; Palmer and Alper 2018). Therefore, numerous studies focused on metabolic engineering of microorganisms towards improved malonyl-CoA availability. In this review article, we provide an overview of advancements in engineering relevant microbial hosts such as *Escherichia coli*, *Saccharomyces cerevisiae*, and *Corynebacterium glutamicum* towards improved malonyl-CoA availability and its effect on product range and productivity.

## Increasing acetyl-CoA supply is essential for improving intracellular malonyl-CoA availability

**Fig. 2:**
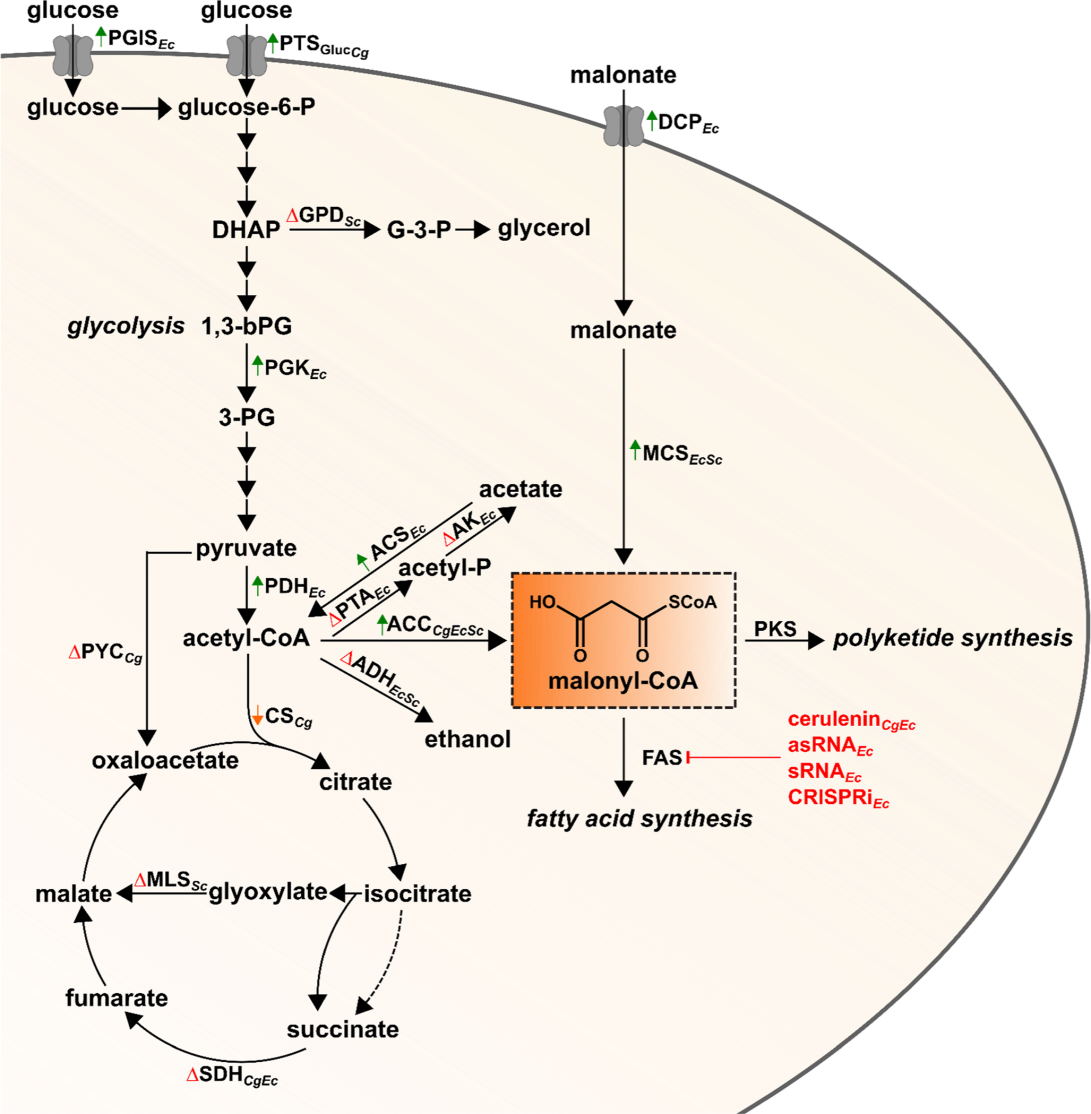
Overview of metabolic engineering strategies towards improved intracellular malonyl-CoA availability in microorganisms. 1,3-bPG 1,3-bisphosphoglycerate, 3-PG 3-phosphoglycerate, ACC acetyl-CoA carboxylase ACS acetyl-CoA synthetase, ADH alcohol dehydrogenase, AK acetate kinase, asRNA antisense RNA, CRISPRi clustered regularly interspaced short palindromic repeats interference, CS citrate synthase, DHAP dihydroxyacetone phosphate, DCP dicarboxylate carrier protein, FAS fatty acid synthase, G-3-P glycerol-3-phosphate, GPD glycerol-3-phosphate dehydrogenase, MCS malonyl-CoA synthetase, MLS malate synthase, PDH pyruvate dehydrogenase complex, PGIS PTS-independent glucose import system, PGK phosphoglycerate kinase, PKS polyketide synthase, PTA phosphate acetyltransferase, PTS_Gluc_ glucose-specific phosphotransferase system, PYC pyruvate carboxylase, SDH succinate dehydrogenase complex, sRNA small regulatory RNA. Increased or heterologous enzyme activity is indicated by an upward green arrow (), reduced enzyme activity by a downward orange arrow (), and eliminated enzyme reactions by a red delta (). Metabolic engineering strategies presented in this review are assigned to the particular microorganism by subscripted abbreviations for *C. glutamicum* (_*Cg*_), *E. coli* (_*Ec*_), and *S. cerevisiae* (_*Sc*_)

Typically, tailoring the central carbon metabolism towards increased availability of acetyl-CoA as direct malonyl-CoA precursor molecule is beneficial for malonyl-CoA synthesis and all malonyl-CoA-derived products. In this context, several metabolic engineering strategies aiming for both improved synthesis and reduced consumption of acetyl-CoA have been developed (Fig. 2).

Recently, it could be shown that focusing the glycolytic flux towards acetyl-CoA by partial elimination of anaplerotic pyruvate carboxylation contributes to the malonyl-CoA-dependent synthesis of noreugenin (53 mg/L), a pentaketide from the medical plant *Aloe arborescens*, in *C. glutamicum* (Milke et al. 2019b). In *E. coli*, increasing flux through the glycolytic pathway by overexpressing genes encoding the phosphoglycerate kinase and pyruvate dehydrogenase enabled the accumulation of naringenin (474 mg/L) (Xu et al. 2011). Also in *E. coli*, increased glucose utilization due to the implementation of an alternative, PTS-system-independent glucose facilitator protein from *Zymomonas mobilis* contributed to an increased acetyl-CoA-dependent synthesis of *N*-acetylglutamate (Zhang et al. 2019). Likewise, deregulated expression of *iolT1* in *C. glutamicum*, encoding a glucose/*myo*-inositol permease, improved glucose uptake, which in turn allowed for increased polyketide and hydroxybenzoic acid synthesis (Brüsseler et al. 2018; Kallscheuer and Marienhagen 2018; Milke et al. 2019b).

In *E. coli*, deletion of acetyl-CoA consuming acetate and ethanol forming pathways resulted in a 15-fold improved malonyl-CoA availability, allowing for the synthesis of phloroglucinol (1280 mg/L) (Fig. 1) (Zha et al. 2009). Besides preventing acetate formation under aerobic conditions from acetyl-CoA, expression of a gene encoding an acetyl-CoA synthetase enabled recycling of acetate to increase the acetyl-CoA pool (Wu et al. 2017). This strategy was applied in *E. coli* to increase acetyl-CoA availability 3.7-fold, ultimately increasing medium chain fatty acid synthesis to 683 mg/L. In *S. cerevisiae*, deletion of genes encoding alcohol dehydrogenases and glycerol-3-phosphate dehydrogenases, partially or completely inhibiting ethanol and glycerol biosynthesis, ultimately improved acetyl-CoA availability two-fold, contributing to the synthesis of over 100 mg/L *n*-butanol in high cell-density fermentations (Lian et al. 2014). Furthermore, inactivation of the alcohol dehydrogenase ADH1 in yeast was demonstrated to increase fatty acid synthesis 1.9-fold reaching titers of up to 120 mg/L (Li et al. 2014).

However, oxidation in the tricarboxylic acid (TCA) cycle or glyoxylate shunt is the main fate of acetyl-CoA. In this context, disruption of the glyoxylate shunt involved in the transport of acetyl-CoA between the cytosol and cellular compartments in *S. cerevisiae* was demonstrated to increase cytosolic acetyl-CoA availability for the biosynthesis of *n*-butanol (Lian et al. 2014). Furthermore, sophisticated metabolic models for *E. coli* and *C. glutamicum* were used to predict multiple gene knock-out combinations, which would increase acetyl-CoA availability. In this context, interruption of the TCA cycle by deleting succinate dehydrogenase complex encoding genes was demonstrated to reduce acetyl-CoA consumption, allowing for the two-fold increased naringenin synthesis in both microorganisms (Fowler et al. 2009; Hartmann et al. 2017; Milke et al. 2019a). However, interrupting the TCA cycle has a negative impact on growth rate and biomass formation on industrially relevant carbon- and energy sources, especially in *C. glutamicum*, which puts this particular modification into question with regard to its overall beneficial impact. Alternatively, to reduce acetyl-CoA consumption via the TCA cycle in *C. glutamicum*, activity of the pace-making citrate synthase (CS) was reduced stepwise to 5.5% compared with the wild-type CS activity by exchanging the native promotor of the CS-encoding *gltA* gene (Milke et al. 2019a, b). Interestingly, overall biomass formation was barely distinguishable from the parental *C. glutamicum* strain with wild-type CS activity, although the growth rate was significantly reduced. Eventually, naringenin synthesis was increased ten-fold to 19 mg/L, indicating an increased intracellular availability of malonyl-CoA, which was later verified by LC-MS/MS quantification.

## High ACC activity improves malonyl-CoA availability

As malonyl-CoA is solely derived from the carboxylation of acetyl-CoA in *E. coli*, *S. cerevisiae*, and *C. glutamicum*, increasing ACC activity is important to make use of the available acetyl-CoA pool. Whereas bacterial and plant chloroplastic ACCs are organized as complexes of distinct dissociable polypeptides, mammalian, fungal, and plant cytosolic ACCs are single polypeptide chains possessing all catalytically active domains (Cronan and Waldrop 2002; Tong 2005). A widely used strategy to increase ACC activity is expression of heterologous genes encoding ACC subunits. Heterologous expression of genes encoding the heterotetrameric ACC and the biotin ligase from *Photorhabdus luminescens* in *E. coli* improved the microbial synthesis of the plant polyphenol pinocembrin seven-fold, allowing for a maximal product titer of 196 mg/L (Leonard et al. 2007). Interestingly, the ACC of *C. glutamicum* requires only two subunits (AccB1, AccD1) instead of four subunits for catalytic activity, rendering this enzyme an often exploited alternative to heterotetrameric ACCs for improving intracellular malonyl-CoA availability (Miyahisa et al. 2005; Gande et al. 2007; Cheng et al. 2016). However, expression of ACC encoding genes was not always successful in terms of microbial polyphenol synthesis (van Summeren-Wesenhagen and Marienhagen 2015). Interestingly, although originating from this particular organism, episomal overexpression of *accBC* and *accD1* hardly increased malonyl-CoA-dependent naringenin biosynthesis in *C. glutamicum* itself (Milke et al. 2019a). Thus, metabolic engineering of *C. glutamicum* to increase transcription of the genome-encoded *accBC* and *accD1* genes could be a more promising strategy for improving malonyl-CoA supply.

Similar to most microorganisms, fatty acid synthesis in *C. glutamicum* is tightly regulated on the transcriptional level (Schujman et al. 2006; Nickel et al. 2010). In the case of *C. glutamicum*, transcription of the ACC genes *accBC* and *accD1*, but also of the FAS encoding genes *fasA* and *fasB*, is inhibited by the TetR-type transcriptional repressor FasR, which binds to a highly conserved *fasO* motif upstream of the regulated open reading frames. Acyl-CoA thioesters (oleoyl-CoA and palmitoyl-CoA) are regarded as effectors interacting with FasR (Irzik et al. 2014). In this context, it was shown that a FasR-S20N mutant, characterized by increased transcription levels of *accD1*, *fasA*, and *fasB*, dramatically improved the production of malonyl-CoA-dependent fatty acids, mainly oleic acid (Takeno et al. 2013). This particular amino acid substitution was postulated to either interfere with FasR-acyl-CoA complex formation or binding of the repressor-effector complex to the *fasO* motifs. Alternatively, in-frame deletion of the FasR-encoding gene *fasR* also increased oleic acid production (Takeno et al. 2013). However, this particular deletion was not beneficial for naringenin synthesis using *C. glutamicum*, probably due to the increased malonyl-CoA consumption by FASs (FasA and FasB), encoded by the FasR-controlled genes *fasA* and *fasB* (Milke et al. 2019a). In a more sophisticated approach, mutation of individual nucleotides within the *fasO* motifs of the *accBC* and *accD1* promoters repealed FasR-mediated regulation, allowing for an almost tripled intracellular malonyl-CoA concentration in *C. glutamicum* (Milke et al. 2019b). The constructed *C. glutamicum* strain did not only enable synthesis of the plant pentaketide noreugenin but also allowed for the formation malonyl-CoA-dependent polyketides 6-methylsalicylic acid (6-MSA) as well as different biotechnologically interesting flavoring phenylbutanoids (Kallscheuer et al. 2019; Milke et al. 2020). In principle, transcriptional deregulation of genomic ACC encoding genes appears to be feasible strategy for all microbial hosts, provided that such a regulation is present. Additionally, omission of auxiliary plasmids for improving ACC activity could not only lower the metabolic burden but also reduce overall process costs (Wu et al. 2016).

In *S. cerevisiae*, ACC activity is not only transcriptionally but also post-translationally regulated. At the transcriptional level, replacement the native promotor of the *ACC1* gene with the strong constitutive *TEF1* promotor improved 6-MSA production yielding up to 554 mg/L in bioreactor cultivations (Wattanachaisaereekul et al. 2008). In terms of post-translational regulation, removal of the Snf1-dependent phosphorylation of Acc1 improved malonyl-CoA supply, allowing for an increased synthesis of fatty acid ethyl esters and 3-hydroxypropionic acid (Shi et al. 2014).

Alternatively, the ACC-independent formation of malonyl-CoA using the malonate assimilation pathway from *Rhizobium trifolii* increased microbial synthesis of the polyphenolic polyketide pinocembrin up to 15-fold (final titer 480 mg/L) using *E. coli* (An and Kim 1998; Leonard et al. 2008; Wu et al. 2013). In *S. cerevisiae*, overexpression of *AAE13*, encoding the malonyl-CoA synthetase from *Arabidopsis thaliana*, significantly improved microbial resveratrol and fatty acid synthesis (Wang et al. 2014). Noteworthy, a temperature-sensitive *ACC1* variant could be complemented by this heterologous pathway.

## Increasing malonyl-CoA availability for polyketide production by inhibition of fatty acid synthesis

In general, the strategies described above are applicable for microbial synthesis of polyketides and fatty acids. However, in terms of microbial polyketide synthesis, fatty acids are regarded as undesired byproducts withdrawing malonyl-CoA and thus limiting overall polyketide formation (Milke et al. 2018). Therefore, endogenous fatty acid synthesis represents a promising target to increase malonyl-CoA availability for polyketide synthesis.

In order to reduce undesired malonyl-CoA consumption, supplementation of the potent FAS inhibitor cerulenin was widely used, which allowed for an increased synthesis of polyphenolic polyketides in *E. coli* and *C. glutamicum* (Leonard et al. 2008; Lim et al. 2011; Santos et al. 2011; van Summeren-Wesenhagen and Marienhagen 2015; Kallscheuer et al. 2016). However, drawback of cerulenin supplementation is the non-selective and irreversible inhibition of FASs by covalently binding to a conserved active site cysteine residue of the KS domain, which almost instantaneously stops microbial growth due to rapid fatty acid depletion (Price et al. 2001). Furthermore, not only the KS domain of FASs but also the KS domain of PKSs could be inhibited, possibly limiting, or even preventing the desired polyketide formation (Ferrer et al. 1999). Additionally, cerulenin is very expensive, rendering application of this antibiotic unsuitable for any large-scale applications (Milke et al. 2019a).

Alternatively, post-transcriptional downregulation of genes involved in fatty acid synthesis in *E. coli* using antisense RNAs, small regulatory RNAs, or CRISPR interference was used to improve malonyl-CoA-dependent synthesis of the polyphenolic polyketides naringenin, resveratrol, and pinosylvin (Wu et al. 2014; Cress et al. 2015; Wu et al. 2015; Yang et al. 2015; Liang et al. 2016; Yang et al. 2018).

## Untargeted strain evolution towards improved malonyl-CoA availability

In addition to the rational metabolic engineering strategies described, undirected approaches involving the generation of random genetic diversity and subsequent screening were also successfully used to isolate microbial strain variants with increased intracellular malonyl-CoA availability. In this context, transcriptional biosensors, linking production phenotypes to fluorescence output signals in combination with fluorescence activated cell sorting (FACS), represent powerful high-throughput tools for rapidly isolating mutants from genetically diverse strain libraries (Flachbart et al. 2019). Available malonyl-CoA-responsive biosensors are based on the transcriptional repressor FapR, which is involved in regulation of fatty acid synthesis in *Bacillus subtilis* (Schujman et al. 2006; Xu et al. 2014b; Johnson et al. 2017). In addition to the construction of synthetic regulatory circuits dynamically controlling gene expression, a FapR-based transcriptional biosensor was used to identify novel gene targets in *S. cerevisiae* for improved malonyl-CoA availability (Xu et al. 2014a; Li et al. 2015; Johnson et al. 2017). In a FACS screening campaign, isolated *S. cerevisiae* variants showing increased expression of the genes *PMP1* and *TPI1* involved in biotin uptake and ATP availability accumulated significantly more 3-hydroxypropionic acid (Li et al. 2015). Alternatively, colorimetric screening assays indicating improved malonyl-CoA availability can be used. For this purpose, the malonyl-CoA-dependent synthesis of the red-colored polyketide flaviolin, which is catalyzed by the type III PKS RppA, found an application (Yang et al. 2018). In the microtiter plate format, this assay was successfully used to screen a synthetic small regulatory RNA library, identifying 14 knockdown targets in *E. coli*. Furthermore, the RppA biosensor was also demonstrated to be functional in *Pseudomonas putida* and *C. glutamicum*. In combination with miniaturized and automated adaptive laboratory evolution experiments, this screening approach represents a promising strategy for the untargeted optimization of rationally engineered strains.

## Data Availability

Not applicable.
